# Progesterone regulates tissue non-specific alkaline phosphatase (TNSALP) expression and activity in ovine utero-placental tissues

**DOI:** 10.1186/s40104-024-01048-x

**Published:** 2024-07-03

**Authors:** Claire Stenhouse, Katherine M. Halloran, Emily C. Hoskins, Robyn M. Moses, Guoyao Wu, Heewon Seo, Gregory A. Johnson, Larry J. Suva, Dana Gaddy, Fuller W. Bazer

**Affiliations:** 1 Department of Animal Science, Pennsylvania State University, University Park, PA 16802 U.S.A.; 2 Department of Animal Science, Texas A&M University, College Station, Texas, 77843 U.S.A.; 3 Department of Pediatrics, University of Michigan, Ann Arbor, MI 48109 U.S.A.; 4 College of Veterinary Medicine, University of Tennessee, Knoxville, TN 37996 U.S.A.; 5 Department of Obstetrics and Gynecology, University of Nebraska Medical Center, Omaha, NE 68198 U.S.A.; 6 Department of Veterinary Integrative Biosciences, Texas A&M University, College Station, Texas, 77843 U.S.A.; 7 Department of Animal and Avian Sciences, University of Maryland, College Park, Maryland, 20742 U.S.A.; 8 Department of Veterinary Physiology and Pharmacology, Texas A&M University, College Station, Texas, 77843 U.S.A.

**Keywords:** Alkaline phosphatase, Phosphate, Placenta, Sheep, Uterus

## Abstract

**Background:**

Tissue non-specific alkaline phosphatase (TNSALP; encoded by the *ALPL* gene) has a critical role in the postnatal regulation of phosphate homeostasis, yet how TNSALP activity and expression are regulated during pregnancy remain largely unknown. This study tested the hypothesis that progesterone (P4) and/or interferon tau (IFNT) regulate TNSALP activity during pregnancy in sheep.

**Methods:**

In Exp. 1, ewes were bred and received daily intramuscular injections of either corn oil vehicle (CO) or 25 mg progesterone in CO (P4) for the first 8 days of pregnancy and were hysterectomized on either Day 9, 12, or 125 of gestation. In Exp. 2, ewes were fitted with intrauterine catheters on Day 7 of the estrous cycle and received daily intramuscular injections of 50 mg P4 in CO and/or 75 mg progesterone receptor antagonist (RU486) in CO from Days 8 to 15, and twice daily intrauterine injections of either control proteins (CX) or IFNT (25 µg/uterine horn/d) from Days 11 to 15 (treatment groups: P4 + CX; P4 + IFNT; RU486 + P4 + CX; and RU486 + P4 + IFNT) and were hysterectomized on Day 16.

**Results:**

In Exp. 1, endometria from ewes administered P4 had greater expression of *ALPL* mRNA than ewes administered CO on Day 12. TNSALP activity appeared greater in the epithelia, stratum compactum stroma, and endothelium of the blood vessels in the endometrium and myometrium from ewes administered P4 than ewes administered CO on Day 12. On Day 125, TNSALP activity localized to uterine epithelial and endothelial cells, independent of P4 treatment. TNSALP activity in placentomes appeared greater in P4 treated ewes and was detected in endothelial cells and caruncular tissue in P4 treated but not CO treated ewes. In Exp. 2, endometrial homogenates from ewes administered RU486 + P4 + CX had lower TNSALP activity those for P4 + CX and P4 + IFNT ewes. Immunoreactive TNSALP protein appeared greater in the mid- and deep-glandular epithelia in RU486 + P4 + CX treated ewes as compared to the other treatment groups. Enzymatic activity appeared greater on the apical surface of the deep glandular epithelia in endometria from ewes treated with RU486 + P4 + CX compared to the other treatment groups.

**Conclusions:**

These results suggest that P4, but not IFNT, regulates the expression and activity of TNSALP in utero-placental tissues and has the potential to contribute to the regulation of phosphate availability that is critical for conceptus development during pregnancy.

**Supplementary Information:**

The online version contains supplementary material available at 10.1186/s40104-024-01048-x.

## Introduction

Across mammalian species, phosphate is transported from the mother to the conceptus (embryo/fetus and associated placental membranes) to allow appropriate regulation of conceptus growth and development [1]. Inorganic phosphate is an essential nutrient with important roles in a wide variety of metabolic pathways including the synthesis of ATP, DNA, and RNA, formation of phospholipids and synthesis of membranes, generation of nucleotides and creatine phosphate, and cell signaling (phosphorylation and dephosphorylation), all of which are key to growth and development of the conceptus [2].

Alkaline phosphatases, critical regulators of phosphate homeostasis postnatally, are membrane-bound glycoproteins that hydrolyze phosphocompound substrates [including phosphoethanolamine (PEA), inorganic pyrophosphate (PPi), and pyridoxal-5′-phosphate (PLP)] to generate phosphate [3, 4]. In humans, four alkaline phosphatase isoenzymes with significant homology are: intestinal (IAP), placental (PLAP), germ cell (GCAP), and tissue non-specific (TNSALP, encoded by the *ALPL* gene). In contrast, sheep have only two known alkaline phosphatase isoenzymes, intestinal-type (IAP) and TNSALP, of which we detected only TNSALP in utero-placental tissues. TNSALP is expressed abundantly in many tissues including liver, bone, and kidney, and has roles in many critical systemic processes including bone mineralization, vitamin B_6_ metabolism, and neurogenesis [4, 5].

We recently characterized: 1) expression of *ALPL* mRNA; 2) localization of TNSALP protein; and 3) quantification and localization of TNSALP enzymatic activity in the endometria of cyclic ewes, and endometria and placentomes at multiple stages of gestation [6]. Interferon tau (IFNT), the maternal signal for pregnancy recognition in ruminants, and progesterone (P4), act both independently and synergistically to alter endometrial expression of mRNAs and proteins important for phosphate transport and utilization during the peri-implantation period of pregnancy in ruminants [7, 8]. We recently demonstrated that the expression of *ALPL* mRNA tended to be lower in endometrium on Day 9 of the estrous cycle compared to Day 1, which could suggest that increasing concentrations of P4 in plasma is associated with decreased endometrial expression of *ALPL* mRNA [6]. Similarly, it has been reported that TNSALP protein is more abundant in ovine cervicovaginal mucus of ewes during estrus compared to diestrus [9]. Further, we recently demonstrated localization of TNSALP protein and enzymatic activity to the apical surface of uterine epithelia (luminal [LE], superficial glandular [sGE] and glandular [GE]) in cyclic and pregnant ewes [6]. As uterine epithelia have important roles in the synthesis and transport of nutrients, it is speculated that TNSALP may convert phosphocompound substrates into phosphate in uterine epithelial cells which could then be used by the epithelial cells or transported into the uterine lumen [6]. Interestingly, in this study we demonstrated significant downregulation in the uterine sGE and deep GE, accompanied by complete absence of TNSALP activity in the uterine LE on Day 17 of pregnancy, which corresponds to the period of conceptus attachment in the sheep.

The intricate and complex interactions during attachment of the elongated conceptus to the apical surface of the uterine LE are critical for implantation and the establishment and subsequent maintenance of a successful pregnancy [10]. The downregulation of progesterone receptor (PGR) in the uterine LE is a conserved process across eutherian mammals which must precede implantation and conceptus attachment to uterine LE/sGE [11]. Following downregulation of PGR in uterine epithelia, uterine LE and sGE express proteins that are either induced by P4 or induced by P4 and further stimulated by IFNT [12–14]. We speculate that downregulation of TNSALP in the uterine LE during the peri-implantation period may be regulated by IFNT, the maternal recognition signal for pregnancy in ruminants, either alone or synergistically with P4 and PGR downregulation in the uterine epithelia. Collectively, the results available in cyclic and pregnant sheep to date suggest a potential role of TNSALP in the regulation of phosphate transport and homeostasis at the maternal-conceptus interface in ruminants. The temporal changes in TNSALP expression and activity in the reproductive tract across the estrous cycle and during early gestation suggest that P4 and IFNT may impact alkaline phosphatase expression and/or activity in utero-placental tissues.

Exp. 1 determined whether exogenous P4 administration during early pregnancy altered expression of *ALPL* mRNA, cell-specific expression of TNSALP protein, and enzymatic activity of TNSALP in endometria on Days 9 and 12 of gestation, and endometrium and placentomes on Day 125 of gestation. Exp. 2 determined whether P4 and IFNT act independently or synergistically to regulate expression of *ALPL* mRNA, cell-specific localization of TNSALP protein, and enzymatic activity of TNSALP in ovine endometria.

## Materials and methods

### Experimental animals and sample collection

All experimental procedures followed the Guide for the Care and Use of Agriculture Animals in Research and Teaching and were approved by the Institutional Animal Care and Use Committee of Texas A&M University.

#### Exp. 1: Characterization of the effect of exogenous P4 on expression of *ALPL* mRNA, cell-specific expression of TNSALP protein, and enzymatic activity of TNSALP in ovine endometria and placentomes

**Fig. 1:**
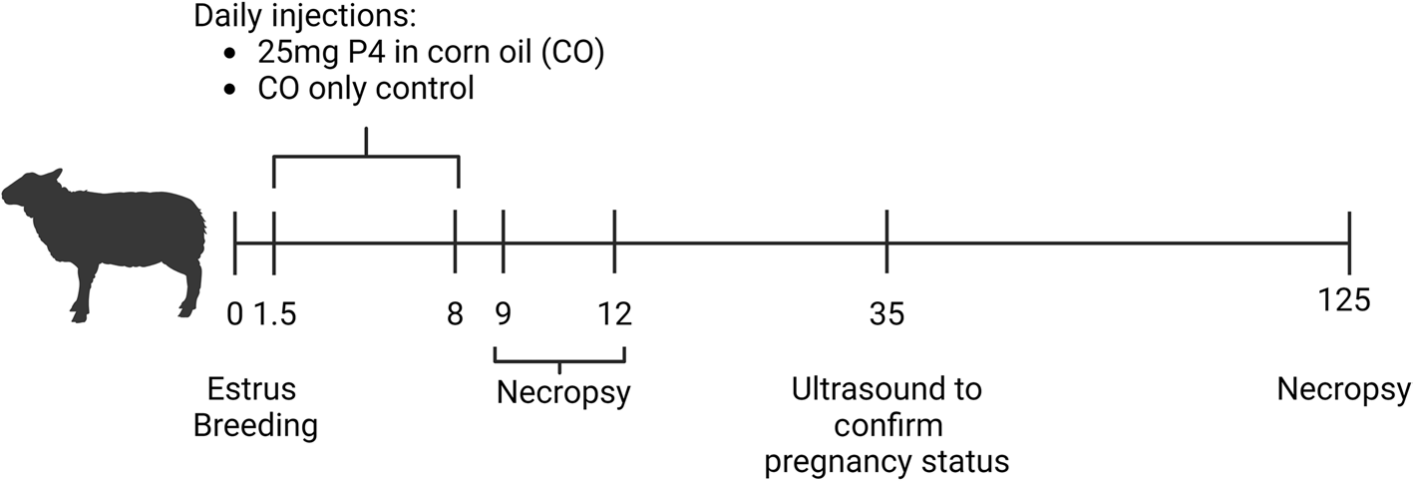
Experiment 1 design. Ewes were assigned randomly to receive daily intramuscular injections of either 25 mg P4 (*n* = 20) in 1 mL corn oil vehicle or 1 mL corn oil alone (CO, *n* = 28) from Day 1.5 (36 h after onset of estrus) through Day 8 of pregnancy. Ewes were euthanized and then hysterectomized on either Day 9 (CO, *n* = 5; P4, *n* = 6), 12 (CO, *n* = 9; P4, *n* = 4), or 125 (CO, *n* = 14; P4, *n* = 10) of gestation

The experimental design and methods for collection of samples in this study is summarized in Fig. 1 and have been published with respect to effects of exogenous P4 supplementation on conceptus development, polyamine synthesis and secretion, and expression of other molecules with suggested roles in phosphate, calcium, and vitamin D signaling [8, 15, 16]. All ewes had exhibited a minimum of two estrous cycles of normal duration (16–18 d) prior to synchronization. Estrous cycles of mature Suffolk ewes were synchronized using a P4 intravaginal insert (CIDR, Zoetis, Parsippany, New Jersey, USA) for 12 d followed by an intramuscular injection of prostaglandin F2α (20 mg Lutalyse, Zoetis) upon CIDR removal. Upon detection of estrus (designated as Day 0), ewes were placed with a fertile ram for 36 h and rams were changed every 12 h during this period. Each ewe was exposed to three rams during estrus, with a total of five rams utilized in the study. Ewes were assigned randomly to receive daily intramuscular injections of either 25 mg P4 (*n* = 20) in 1 mL corn oil vehicle or 1 mL corn oil alone (CO, *n* = 28) from Day 1.5 (36 h after onset of estrus) through Day 8 of pregnancy. Ewes were euthanized and then hysterectomized on either Day 9 (CO, *n* = 5; P4, *n* = 6), 12 (CO, *n* = 9; P4, *n* = 4), or 125 (CO, *n* = 14; P4, *n* = 10) of gestation. Day 9 was selected as this corresponds to the period of elevated production of P4 by the corpus luteum during the preimplantation period of pregnancy in the sheep. Downregulation of PGR expression in endometrial LE and sGE in a normal pregnancy occurs by Days 12–13 of gestation in sheep and is an important prerequisite for conceptus attachment. Exogenous progesterone supplementation in early pregnancy, advances the downregulation of PGR and enhances conceptus development on both Days 9 and 12 of gestation [16]. Day 125 was selected as this corresponds to the period of exponential fetal growth.

On Days 9 and 12, the uterine horns were flushed with 10 mL of sterile phosphate buffered saline (PBS, pH 7.2) into a grid dish. Pregnancy status was confirmed by the presence of a normally developed conceptus. On Day 9, blastocysts from P4-treated ewes (0.025 ± 0.015 mm^3^) had greater volumes than blastocysts (0.013 ± 0.004 mm^3^) from CO-treated ewes [16]. All conceptuses recovered from P4-treated ewes on Day 12 were elongated and filamentous while conceptuses from CO-treated ewes were spherical [16]. These findings suggest that conceptus development was accelerated on both Days 9 and 12 of gestation in response to exogenous P4. The uterus was opened along the mesometrial side, and endometrial samples were taken from the anti-mesometrial side of the uterine horn ipsilateral to the corpus luteum. Endometrial samples from Days 9 and 12 of pregnancy were either frozen in liquid nitrogen and stored at −80 °C or fixed in 4% parafrmaldehyde and stored in 70% ethanl prior to embedding in paraffin wax.

On Day 125, litter size was characterized by the number of fetuses (singleton vs. twin) present at the time of hysterectomy (*n* = 5 CO singles, 9 CO twins, 5 P4 singles, and 5 P4 twins). The chorioallantois was separated from the endometrium to expose the fetus and placental membranes. Sections of intercaruncular endometrium and whole placentomes were collected from the uterine horn ipsilateral to the corpus luteum and frozen in liquid nitrogen and stored at –80 °C or fixed in 4% paraformaldehyde and stored in 70% ethanol prior to embedding in paraffin.

#### Exp. 2: Characterization of the effect of P4 and IFNT on expression of ALPL mRNA, cell-specific immunolocalization of TNSALP protein, and enzymatic activity of TNSALP in ovine endometria

**Fig. 2:**
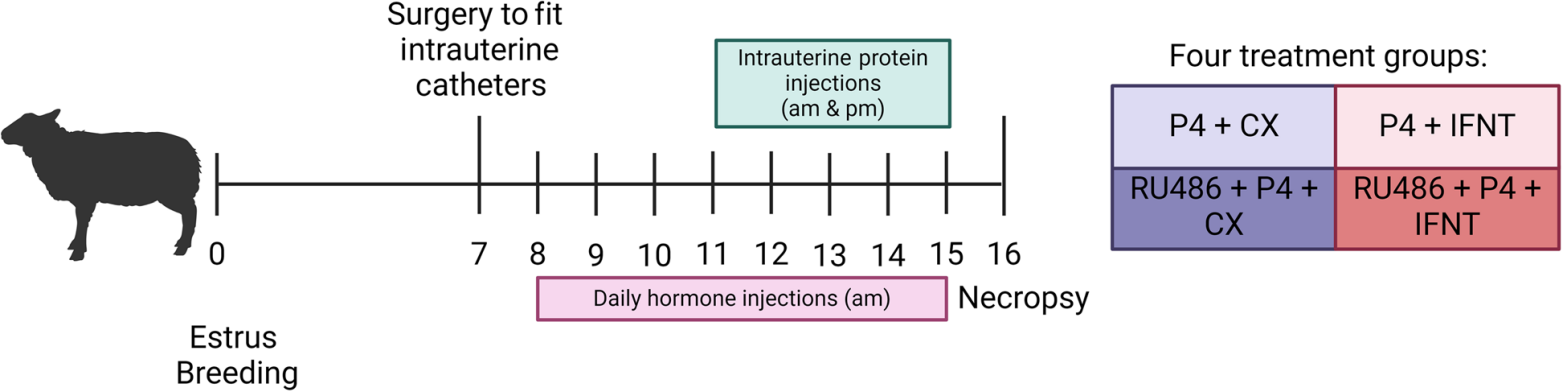
Experiment 2 design. Catheters were surgically fitted into the uteri on Day 7 of the estrous cycle. From Day 8 of the estrous cycle, ewes were assigned randomly to receive daily intramuscular injections of 50 mg progesterone (P4) dissolved in ethanol and suspended in corn oil vehicle, or 50 mg P4 and 75 mg progesterone receptor antagonist (mifepristone, also known as RU486, Sigma Aldrich; M8046, [P4 + RU486]), which was dissolved in ethanol and suspended in corn oil vehicle. From Day 11 of the estrous cycle, ewes received twice daily intrauterine injections via intrauterine catheters of either control serum proteins (25 µg/uterine horn/d [CX]) or IFNT (25 µg/uterine horn/d [IFNT]). This resulted in a 2 × 2 factorial design: 1) P4 + CX (*n* = 6); 2) P4 + IFNT (*n* = 6); 3) RU486 + P4 + CX (*n* = 5); and 4) RU486 + P4 + IFNT (*n* = 6)

The experimental design and methods for collection of samples in this study are summarized in Fig. 2 have been published with respect to the regulatory effects of P4 and IFNT on endometrial synthesis and secretion of polyamines [17] and the expression of other molecules with important regulatory roles for phosphate, calcium, and vitamin D signaling [7]. In brief, estrous cycles of mature Rambouillet ewes (*n* = 23) were synchronized as described for Exp. 1. Following CIDR removal and administration of prostaglandin F2α, ewes were observed for estrus (designated as Day 0) in the presence of a vasectomized ram. Ewes were surgically fitted with bilateral intrauterine catheters on Day 7 of the estrous cycle as described previously [18]. A surgical plane of anesthesia was induced and maintained with Isoflurane for ewes to allow exposure of the reproductive tract via mid-ventral laparotomy. Catheters were inserted into the lumen of both uterine horns, approximately 2 cm below the utero-tubal junction. The catheters were exteriorized through the flank, and the external portion of the catheters stored in a plastic bag within a cloth pouch sutured to the skin. Catheters were cleared with saline and capped with a sterilized nail when not in use.

Recombinant ovine IFNT (1 × 10^8^ antiviral units) was prepared [19] for delivery into the uterine lumen via the intrauterine catheters by diluting stock IFNT (1.5 mg/mL) to deliver 12.5 µg IFNT in 1 mL of sterile PBS per uterine horn twice daily (total of 25 µg/uterine horn/d). To generate control serum proteins, a jugular blood sample was collected from a mature ram. Serum was collected by centrifugation at 3,000 × *g* for 30 min at 4 °C, filtered (0.45 μm), and stored at –20 °C until used. The concentration of total protein in the serum was quantified spectrophotometrically (Spectramax M2, Molecular Devices, San Jose, CA, USA; absorbance 562 nm) using protein assay dye reagent (BioRad, Hercules, CA, USA; 500-0006) according to the manufacturer’s instructions. Serum proteins were diluted in saline to 12.5 µg/mL and 1 mL was delivered into each uterine horn twice daily (total of 25 µg/horn/d).

On Day 8 of the estrous cycle, ewes were assigned randomly to receive daily (0700 h) intramuscular injections of 50 mg P4 (Sigma Aldrich, St. Louis, MO, USA; P8783), dissolved in ethanol and suspended in corn oil vehicle, or 50 mg P4 and 75 mg progesterone receptor antagonist (mifepristone, also known as RU486, Sigma Aldrich; M8046, [P4 + RU486]), which was dissolved in ethanol and suspended in corn oil vehicle. From Day 11 of the estrous cycle, ewes received twice daily (0700 h and 1700 h) intrauterine injections via intrauterine catheters of either control serum proteins (25 µg/uterine horn/d [CX]) or IFNT (25 µg/uterine horn/d [IFNT]). This resulted in a 2 × 2 factorial design: (1) P4 + CX (*n* = 6); (2) P4 + IFNT (*n* = 6); (3) RU486 + P4 + CX (*n* = 5); and (4) RU486 + P4 + IFNT (*n* = 6). Intrauterine injections of proteins were followed by 50 mg of ampicillin (Gibco, Grand Island, NY, USA) in 0.1 mL of saline followed by 0.9 mL saline to clear the catheter.

Ewes were euthanized and hysterectomized on Day 16 of the estrous cycle. Day 16 was selected as this corresponds to the period of maximal IFNT production by the conceptus in the pregnant sheep [20]. Cross sections of intact uteri were fixed overnight in 4% praformaldehyde and stored in 70% ehanol prior to embedding in paraffin wax. Endometrium was dissected from the myometrium of the uterus, frozen in liquid nitrogen, and stored at –80 °C.

### Quantification of *ALPL* mRNA expression by qPCR

The relative expression of *ALPL* mRNA was quantified by qPCR as described previously [6]. RNA was extracted from snap-frozen endometria (Exp. 1 and 2), and placentomes (Exp. 1) as described previously [7]. The RNA was quantified spectrophotometrically (NanoDrop ND-1000 Spectrophotometer), and all samples had a A_260_/A_280_ value greater than 2. Complementary DNA (cDNA) was synthesized from RNA with SuperScript II reverse transcriptase and oligo (deoxythymidine) primers (Invitrogen, Carlsbad, CA, USA), as per the manufacturer’s instructions. Negative controls without reverse transcriptase were included to test for genomic DNA contamination and all cDNA was stored at –20 °C until required. Quantitative polymerase chain reaction (qPCR) was performed using the ABI prism 7900HT system (Applied Biosystems, Foster City, CA, USA) with Power SYBR Green PCR Master Mix (Applied Biosystems), instructions to determine expression of candidate mRNAs as described previously [7]. Primer sequences are provided in Additional file 1. The stability of reference genes was assessed by geNORM V3.5 (Ghent University Hospital, Centre for Medical Genetics, Ghent, Belgium) in each tissue, with an M value of < 1.5 considered stable. Reference genes used for each experiment are detailed in Additional file 1. No Day and/or treatment effects were detected for the reference genes utilized, determined using GenStat (Version 13.1; VSN International Ltd.). The abundances of the candidate mRNAs in the samples were quantified using the ΔΔCq method.

### Immunohistochemistry

 Cell-specific immunohistochemical localization for TNSALP protein in uteri and placentomes was performed using paraffin sections as described previously [6]. In brief, sections (5 μm) were deparaffinized in CitriSolv (Decon Laboratories Inc., Prussia, PA, USA) and rehydrated through a graded series of ethanol to double distilled water. Heat-induced epitope retrieval was performed in Tris-buffer (pH 9.0; Vector Laboratories, Burlinghame, California, USA). Endogenous peroxidase activity was blocked by incubation with 0.3% hydrogen peroxide (Sigma Aldrich) in methanol, and non-specific binding sites were blocked by incubation with normal horse serum (Vectastain Elite Universal ABC kit; Vector Laboratories) for 1 h at room temperature. Sections were incubated with a primary antibody (11187, Proteintech, Rosemont, IL, USA; 1.5 µg/mL) or with rabbit immunoglobulin G (rIgG; 1.5 µg/mL; Vector Laboratories). The slides were incubated overnight in a humidified chamber at 4 °C, washed in PBS, and incubated for 1 h at 37 °C in a humidified chamber with a biotinylated anti-rabbit IgG secondary antibody (Vectastain Elite ABC kit; Vector Laboratories) at 0.005 mg/mL in PBS containing 1.5% normal horse serum. Sections were incubated with Vectastain Elite ABC reagent (Vectastain Elite ABC kit; Vector Laboratories) for 30 min at 37 °C in a humidified chamber. Slides were washed in PBS followed by a wash in 0.05 mol/L Tris-HCl and incubated with diaminobenzidine-tetra-hydrochloride hydrate (Sigma Aldrich) in 0.05 mol/L Tris-HCl containing hydrogen peroxide. Sections were counterstained with hematoxylin and dehydrated in a graded series of ethanol and CitriSolv (Decon Laboratories Inc.) before coverslips were affixed using Permount mounting medium (Fisher Scientific). Digital images of representative fields from 6 non-overlapping areas per section were recorded under brightfield illumination using a Nikon Eclipse microscope and NIS-Elements AR 4.30.02 64-bit Software (Nikon Instruments Inc, Melville, New York, USA).

### Quantification of TNSALP enzymatic activity in homogenates

Snap-frozen endometrial and placentome samples (~ 100 mg of tissue) were homogenized in 1 mL of lysis buffer (60 mmol/L Tris-HCl (Sigma Aldrich, St. Louis, Missouri, USA), 1 mmol/L Na_3_VO_4_ (Fisher Scientific, Waltham, Massachusetts, USA), 10% glycerol (Fisher Scientific), 1% sodium dodecyl sulfate (BioRad, Hercules, California, USA), containing an EDTA-free protease inhibitor (Roche, Indianapolis, Indiana, USA). Homogenates were centrifuged at 14,000 × *g* for 15 min at 4 °C, and the supernatant was removed and stored at –80 °C until assayed. The concentrations of total proteins in the tissue homogenates were quantified spectrophotometrically (Synergy H1, BioTek, Shoreline, Washington, USA) using a protein assay dye reagent (BioRad; 500-0006) according to the manufacturer’s instructions. The enzymatic activity of TNSALP in tissue homogenates was quantified by a colorimetric enzymatic activity assay (ab83369, Abcam, Waltham, MA, USA) as per the manufacturer’s instructions and the absorbance quantified spectrophotometrically (SynergyH1, BioTek; Abs 405). Enzymatic activity was expressed relative to the protein concentration of the tissue homogenate.

### Localization of TNSALP enzymatic activity

TNSALP enzymatic activity was localized in paraffin-embedded tissue sections (5 μm) as previously described [6]. In brief, tissue sections were deparaffinized in CitriSolv (Decon Laboratories Inc.) and rehydrated through a graded series of ethanol to double distilled water. Sections were incubated with alkaline phosphatase substrate (SK-5300; Vector Laboratories) in 100 mmol/L Tris-HCl (pH 8.3) for 3 h at room temperature. As a negative control, sections were incubated with only 100 mmol/L Tris-HCl. Slides were counterstained for 5 min in 0.5% methyl green in 0.1 mol/L sodium acetate, washed in 95% ethanol, rehydrated, and mounted (ab104131; Abcam).

### Statistical analysis

All statistical analyses were performed using GenStat 13.1 or Graphpad Prism. Mean values were calculated for each individual sample for each parameter investigated and the normality of distribution of the data was assessed using the Anderson–Darling test (*P* > 0.05). Outliers identified by a Robust regression and Outlier removal (ROUT) test were excluded. Transformations were carried out if necessary to achieve a Gaussian distribution. Outliers identified by a ROUT outlier test were excluded. ANOVA with a Tukey post-hoc analysis was performed for analysis of temporal changes across pregnancy in Exp. 1 and treatment effects in Exp. 2. Two-way ANOVA for 1) Day × treatment and 2) treatment × litter size was performed. The results were considered significant at *P* < 0.05.

## Results

### Exp. 1: Characterization of the effect of exogenous P4 on the expression of *ALPL* mRNA, localization of TNSALP protein, and enzymatic activity of TNSALP in ovine endometria and placentomes

#### Endometria

**Fig. 3:**
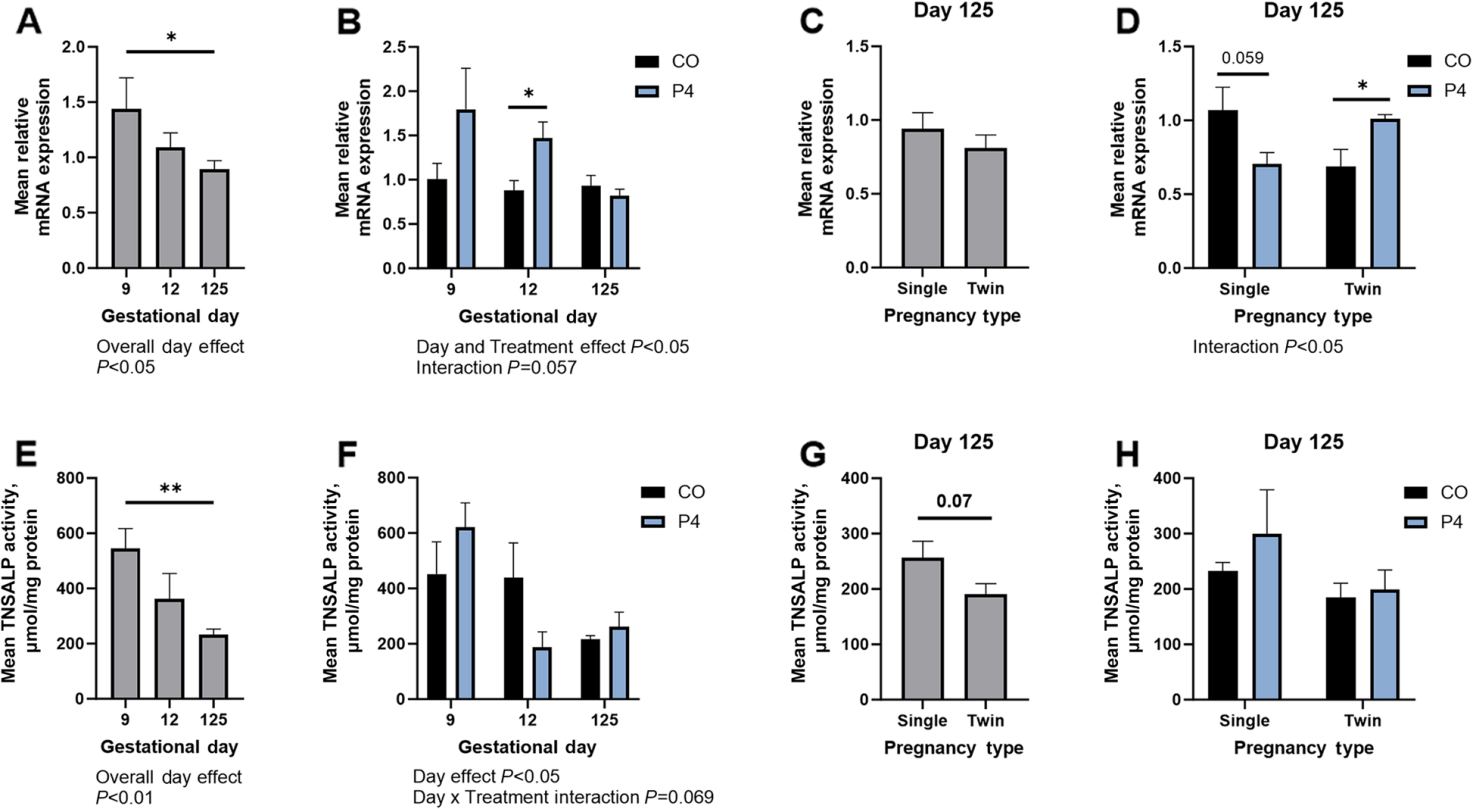
Quantification of expression of *ALPL* mRNA (**A**–**D**) and TNSALP activity (**E**–**H**) in endometria from ewes treated with either corn oil (CO) or progesterone (P4) on Days 9, 12, and 125 of gestation. Mean values presented ± SEM. ^*^P < 0.05. ^**^P < 0.01

Day of gestation and P4 treatment significantly affected endometrial expression of *ALPL* mRNA (*P* < 0.05; Fig. 3A and B). Expression of *ALPL* mRNA was lower on Day 125 of gestation compared to Day 9 (*P* < 0.05). Endometria from P4 treated ewes had greater expression of *ALPL* mRNA compared to CO pregnancies on Day 12 of gestation (*P* < 0.05), with a Day × Treatment interaction that trended towards significance (*P* = 0.08. Fig. 3B). On Day 125 of gestation, endometrial expression of *ALPL* mRNA was not influenced by number of fetuses (Fig. 3C). However, there was a significant interaction between litter size and P4 treatment on the expression of *ALPL* mRNA by the endometrium on Day 125 (*P* < 0.05; Fig. 3D). P4 treatment tended to decrease expression of *ALPL* mRNA in endometria from singleton pregnancies on Day 125 (*P* = 0.059; Fig. 3D). In contrast, P4 treatment increased the expression of *ALPL* mRNA in endometria from twin pregnancies compared to singleton pregnancies on Day 125 (*P* < 0.05; Fig. 3D).

Day of gestation significantly affected endometrial TNSALP activity (*P* < 0.05; Fig. 3E), with decreased enzymatic activity in endometria on Day 125 of gestation compared to Day 9 (*P* < 0.01). P4 treatment did not affect TNSALP activity in endometrial homogenates although there was a tendency towards a significant Day × Treatment interaction (*P* = 0.069; Fig. 3F). There tended to be an effect of litter size on endometrial homogenate TNSALP activity, with endometria from twin pregnancies having less TNSALP activity when compared to that for singleton pregnancies (*P* = 0.07; Fig. 3G). There was no effect of treatment or litter size on TNSALP activity in Day 125 endometria (Fig. 3H).

**Fig. 4:**
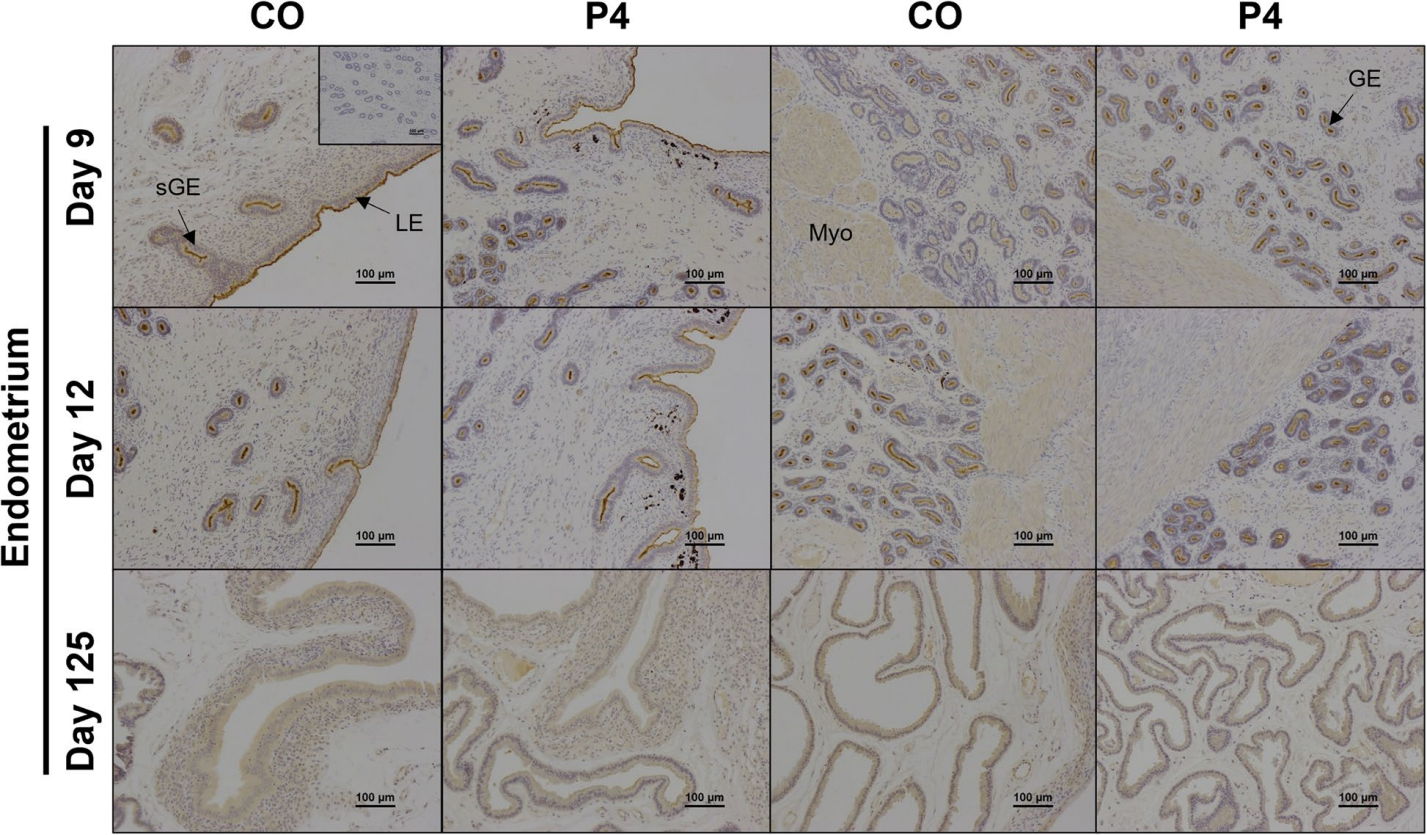
Representative images of TNSALP protein immunolocalization in endometria of pregnant ewes on Days 9, 12, or 125 of gestation. Ewes were treated with either corn oil (CO) or progesterone (P4). Rabbit IgG controls were included at an equivalent concentration of protein to that of the TNSALP antibody as a negative control for immunohistochemical staining (representative image included as an inset image). LE = luminal epithelium, GE = glandular epithelium, sGE = superficial glandular epithelium, Myo = myometrium. Scale bars represent 100 µm. Ewes were euthanized and then hysterectomized on either Day 9 (CO, *n* = 5; P4, *n* = 6), 12 (CO, *n* = 9; P4, *n* = 4), or 125 (*n* = 5 CO singles, 9 CO twins, 5 P4 singles, and 5 P4 twins)

Strong immunoreactivity for TNSALP protein was present at the apical surface of endometrial luminal (LE), glandular (GE), superficial glandular (sGE) epithelia, and myometrium on Days 9 and 12 of gestation (Fig. 4). Weak immunoreactivity for TNSALP protein was present in the endometrial stroma and within the lumen of the blood vessels on Days 9 and 12 of gestation. On Day 125 of gestation, immunoreactivity for TNSALP protein was present in all endometrial epithelia, stratum spongiosum and compactum stroma, endothelial cells, and within the lumen of blood vessels (Fig. 4). Exogenous P4 did not appear to influence TNSALP protein localization or immunoreactivity.

**Fig. 5:**
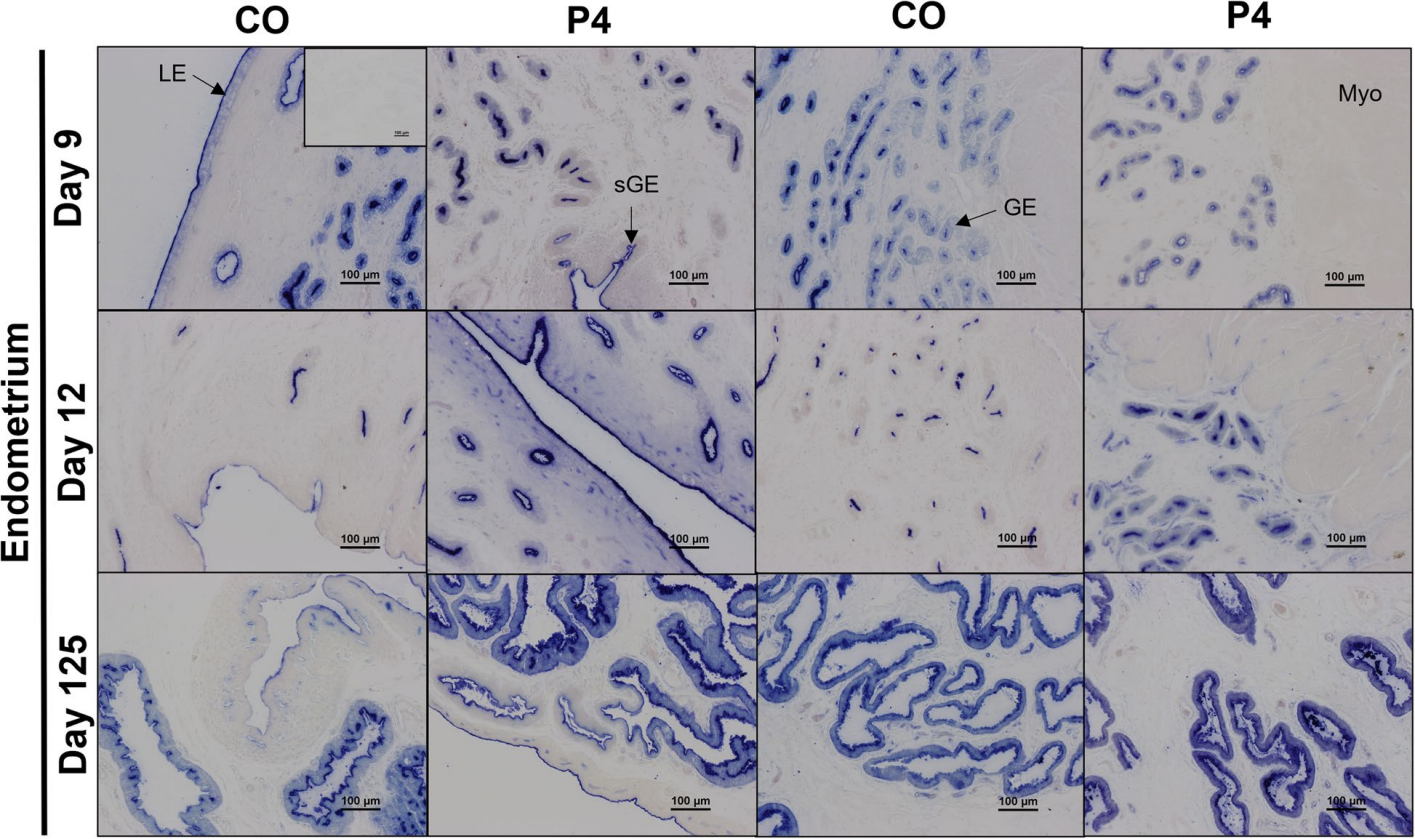
Representative images of the cell-specific localization of TNSALP enzymatic activity in endometria from ewes on Days 9, 12, or 125 of gestation. Ewes were treated with either corn oil (CO) or progesterone (P4). Representative image of negative controls included as an inset image. LE = luminal epithelium, GE = glandular epithelium, sGE = superficial glandular epithelium, Myo = myometrium. Scale bars represent 100 µm. Ewes were euthanized and then hysterectomized on either Day 9 (CO, *n* = 5; P4, *n* = 6), 12 (CO, *n* = 9; P4, *n* = 4), or 125 (CO, *n* = 14; P4, *n* = 10) of gestation

TNSALP enzymatic activity was very abundant at the apical surface of the endometrial epithelia throughout gestation (Fig. 5) and decreased in endometrial LE and GE in endometria from CO ewes between Days 9 and 12. TNSALP activity appeared to be greater in the endometrial epithelia, stratum compactum stroma, and endothelial cells of blood vessels in the endometrium and myometrium of P4 treated ewes compared to CO treated ewes on Day 12. On Day 125 of gestation, TNSALP activity localized to the epithelial cells and endothelial cells, with no apparent effect of P4 treatment on localization.

#### Placentomes

**Fig. 6:**
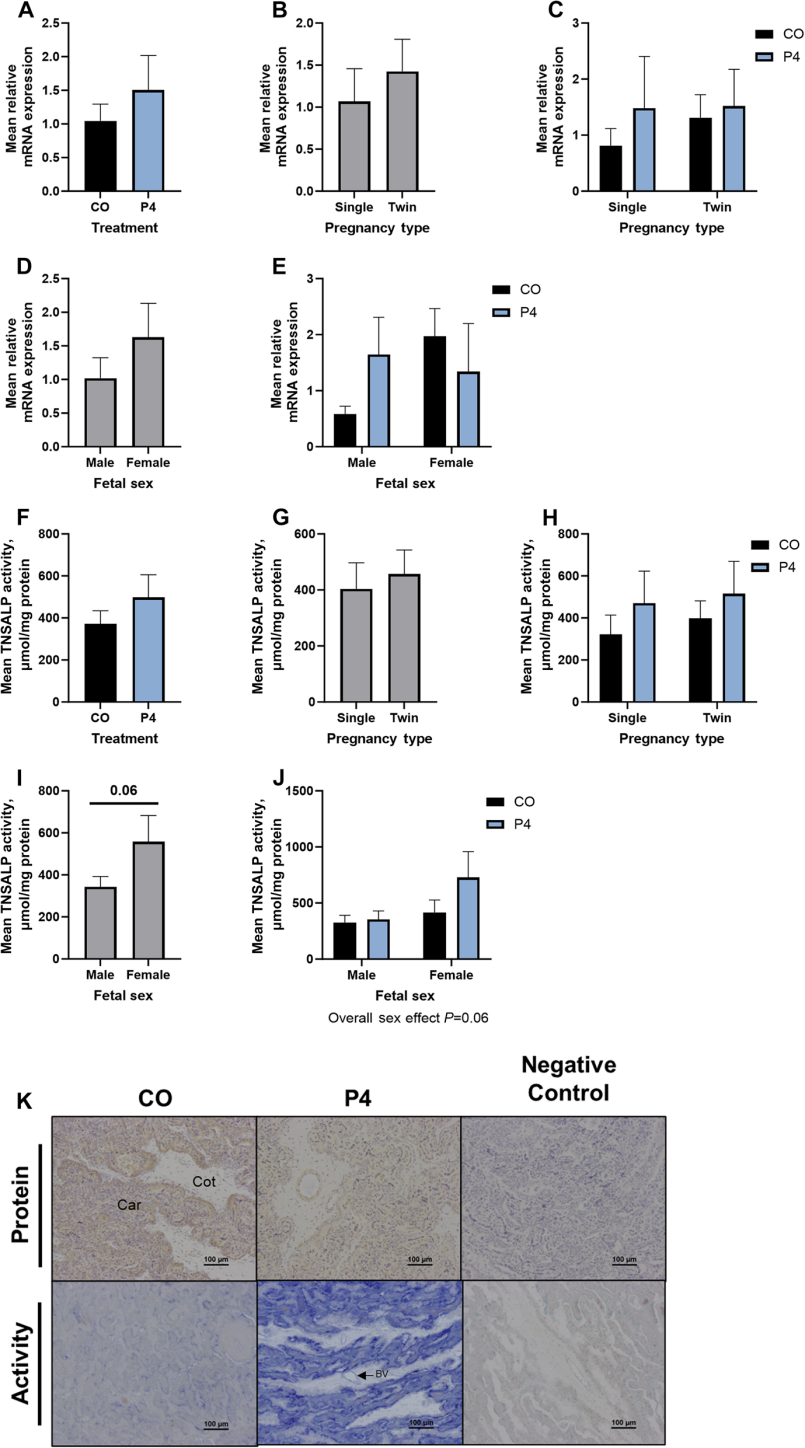
Quantification of expression of *ALPL* mRNA (**A**–**E**) and TNSALP activity (**F**–**J**) in placentomes of ewes treated with either corn oil (CO) or progesterone (P4). Representative images of cell-specific localization of TNSALP protein and activity in placentomes (**K**). Rabbit IgG (RIgG) controls were included at a protein concentration equivalent to that for the TNSALP antibody as a negative control for the immunohistochemical staining. Mean values presented ± SEM. Scale bars represent 100 µm. Car = caruncle, Cot = cotyledon, BV = blood vessel. Ewes were euthanized and then hysterectomized on either Day 9 (CO, *n* = 5; P4, *n* = 6), 12 (CO, *n* = 9; P4, *n* = 4), or 125 (*n* = 5 CO singles, 9 CO twins, 5 P4 singles, and 5 P4 twins) of gestation

Exogenous P4 treatment, litter size, and fetal sex had no effect on the expression of *ALPL* mRNA in Day 125 placentomes (Fig. 6). Similarly, exogenous P4 treatment and litter size had no effect on TNSALP activity in homogenates of placentomes from Day 125 of gestation (Fig. 6F–H). Homogenates of placentomes associated with female fetuses tended to have greater TNSALP activity compared to those associated with male fetuses (*P* = 0.06; Fig. 6I).

TNSALP protein was detected throughout the placentome, with immunoreactivity detected in the chorionic epithelium, cotyledonary and caruncular stroma, syncytium, and tunica muscularis of blood vessels (Fig. 6K). Ewes treated with P4 appeared to have a moderate decrease in TNSALP protein immunoreactivity in placentomes, particularly in the caruncular tissue and along the syncytium.

In placentomes from CO treated ewes, TNSALP enzymatic activity localized to syncytial cells and apical surfaces of the chorionic epithelium. The enzymatic activity in the placentome appeared to increase substantially in response to P4 treatment, with abundant activity detected in endothelial cells of blood vessels and caruncular tissues (Fig. 6K).

### Exp. 2: Characterization of the effect of P4 and IFNT on expression of *ALPL* mRNA, localization of TNSALP protein, and enzymatic activity of TNSALP in ovine endometria

**Fig. 7:**
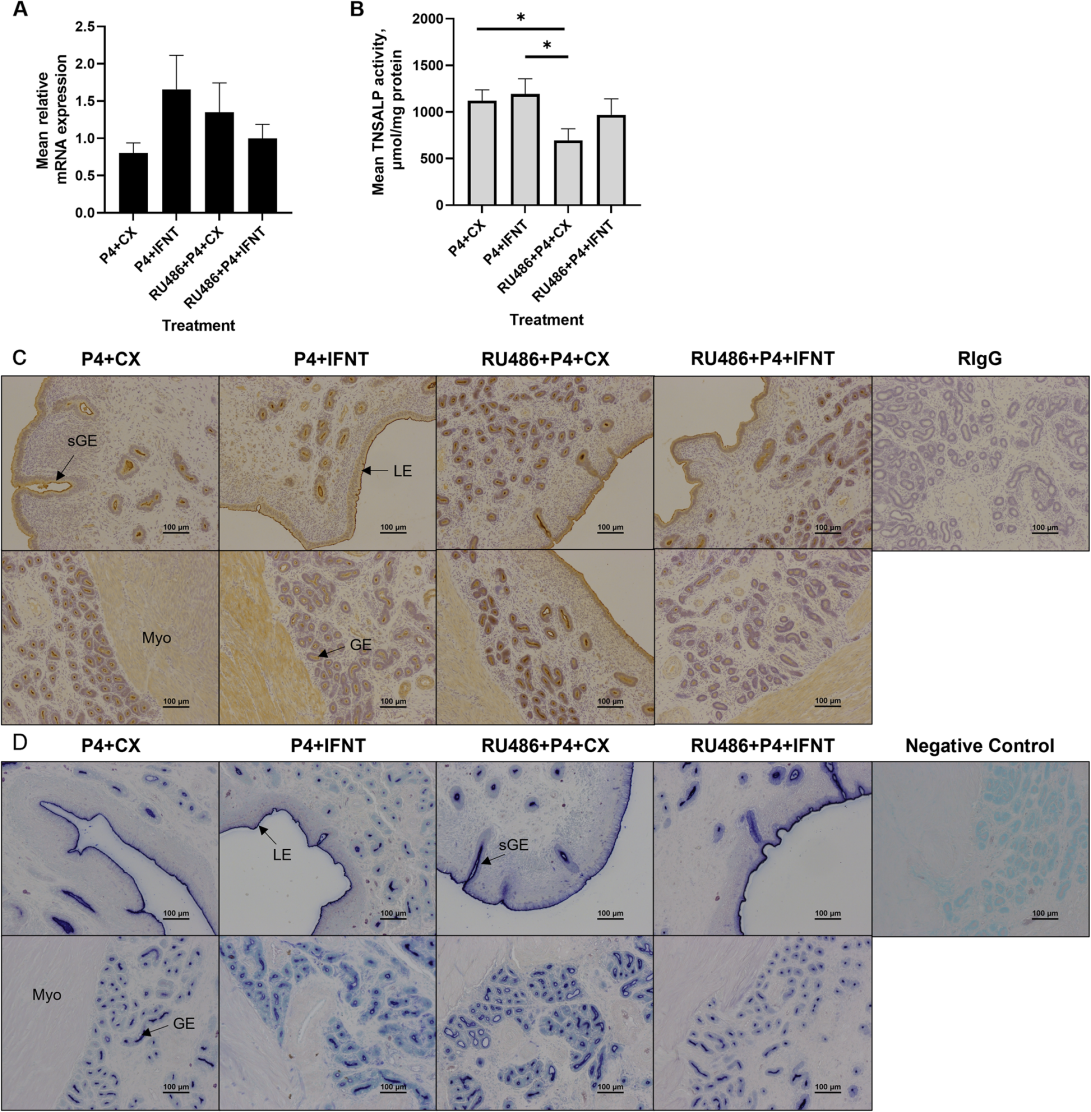
Quantification of expression of *ALPL* mRNA (**A**) and TNSALP activity (**B**), and cell-specific localization of TNSALP protein (**C**) and enzymatic activity (**D**) in endometria from ewes treated with progesterone (P4) + control proteins (CX), P4 + interferon tau (IFNT), mifepristone (RU486) and P4 + CX, and RU486 + P4 + IFNT. Rabbit IgG (rIgG) controls were included at a protein concentration equivalent to that for the TNSALP antibody as a negative control for the immunohistochemical staining. LE = luminal epithelium, GE = glandular epithelium, sGE = superficial glandular epithelium, Myo = myometrium. Scale bars represent 100 μm. P4 + CX (*n* = 6); P4 + IFNT (*n* = 6); RU486 + P4 + CX (*n* = 5); and RU486 + P4 + IFNT (*n* = 6)

Expression of *ALPL* mRNA was not affected by protein or hormonal treatment (Fig. 7A; *P* > 0.10). Homogenates of endometria from ewes treated with RU486 + P4 + CX had lower TNSALP activity than that from ewes treated with P4 and CX proteins and P4 and IFNT (*P* < 0.05; Fig. 7B).

Strong immunoreactivity for TNSALP protein was present at the apical surface of endometrial LE, GE, and sGE (Fig. 7C). Further, TNSALP protein immunolocalized to the myometrium, endothelial cells and tunica muscularis of blood vessels, and weak immunoreactivity was observed in stromal tissue. Immunoreactive TNSALP protein appeared to increase in the mid- and deep-GE in ewes treated with RU486 + P4 + CX compared to the other treatment groups. In contrast, a decrease in TNSALP protein was observed in the deep GE of endometria from ewes treated with RU486 + P4 + IFNT compared to the other treatment groups.

There was significant TNSALP enzymatic activity on the apical surface of the endometrial epithelia of endometria from ewes in all treatment groups (Fig. 7D). Enzymatic activity appeared to be greater on the apical surface of the deep GE in endometria from ewes treated with RU486 + P4 + CX compared to the other treatment groups.

## Discussion

Despite evidence suggesting a link between phosphate and pregnancy outcome [21, 22], the mechanisms regulating phosphate transport at the maternal-conceptus interface remain under-investigated and poorly understood. We recently reported abundant expression and activity of TNSALP in ovine utero-placental tissues throughout gestation [6]. The spatio-temporal changes in TNSALP expression and activity suggested that utero-placental TNSALP expression and activity is regulated by endocrine or paracrine signaling. This study was focused on determining the influence of P4 and IFNT on *ALPL* mRNA expression, TNSALP protein immunolocalization, and enzymatic activity in ovine endometria and placentomes.

P4 is an essential regulator of uterine functions required for the establishment and maintenance of pregnancy in eutherian mammals and has important regulatory roles in both epithelia and stromal cells in the uterus. Temporal and cell-specific downregulation of PGR expression is required for implantation and the establishment of pregnancy in mammals. Downregulation of PGR expression in endometrial LE and sGE occurs by Days 12–13 of gestation in sheep, which alters histotrophic secretions. In sheep, pigs, and cattle, exogenous P4 administered in early pregnancy advances time of down-regulation of PGR in epithelia coincident with the period of accelerated conceptus development [16, 23–30]. In this study, exogenous P4 supplementation from Day 1.5 (36 h after onset of estrus) through Day 8 of pregnancy resulted in larger spherical blastocysts on Day 9, and filamentous conceptuses compared to spherical blastocysts in control ewes on Day 12, which was accompanied by earlier downregulation of PGR in endometrial LE and sGE [16]. In addition to the described effects of P4 on the endometrial epithelia, P4 regulates the expression of growth factors by ovine uterine stroma. For example, it is hypothesized that P4 induces the production of ‘progestamedins’ including fibroblast growth factor (FGF)7, FGF10, and hepatocyte growth factor (HGF), by uterine stromal cells which, via their receptors, act in a paracrine manner to allow P4 to mediate protein expression and function in uterine epithelial cells [31]. Thus, P4 actions on stromal cells may regulate histotrophic secretions and therefore nutrient availability for the conceptus. On Day 12 of gestation, endometria from P4 treated ewes had greater expression of *ALPL* mRNA, which was accompanied by an apparent increase in TNSALP activity in epithelia, stratum compactum stroma, and endothelial cells of blood vessels in the endometrium and myometrium. We have recently reported greater TNSALP activity in the endometrial stratum compactum stroma of Day 14 cycle ewes compared to that for endometria from Day 1 or Day 9 of the estrous cycle [32]. Thus, it could be speculated that following PGR downregulation in the endometrial LE and sGE, P4 not only alters TNSALP to alter phosphate availability in epithelia, but also regulates TNSALP activity in the endometrial stratum compactum stroma, which could alter phosphate availability.

The relationship between P4 and both uterine function and conceptus development during early pregnancy has been studied extensively in ruminants [33–35]. While exogenous P4 supplementation in early gestation advances conceptus development in early pregnancy in ruminants [16, 23–25, 27–30], the long-term effects of exogenous P4 supplementation on conceptus development is poorly understood. In this study, P4 treatment in early pregnancy tended to decrease the expression of *ALPL* mRNA in endometria from singleton pregnancies on Day 125. In contrast, P4 treatment increased the expression of *ALPL* mRNA in endometria from ewes with twin pregnancies on Day 125. Recently, greater expression of other phosphate regulatory molecules was observed in utero-placental tissues on Day 125 of pregnancy for P4-treated ewes with twin pregnancies which was not observed for ewes with singleton pregnancies [8]. Similarly, ewes treated with P4 had a moderate decrease in TNSALP protein expression in placentomes. In contrast, TNSALP enzymatic activity in placentomes increased substantially in response to P4 treatment, with activity detected in the endothelial cells of blood vessels and in the caruncular tissue which was not detected in placentomes of ewes treated with CO. These findings suggest that P4-treatment not only accelerates conceptus development during the peri-implantation period of pregnancy but may have effects on the phosphate signaling pathways in endometria in late gestation, particularly in ewes with twin pregnancies with increased demands for nutrient transport at the maternal-conceptus interface in late gestation [36].

P4 signaling through PGR is critical for the establishment and maintenance of pregnancy, and the regulation of histotrophic secretions by the ovine uterus. It has also been suggested that IFNT, the maternal signal for pregnancy recognition in ruminants, and P4 can act both independently and synergistically to alter endometrial expression of mRNAs and proteins important for phosphate transport and utilization during the peri-implantation period of pregnancy [7, 8]. Exp. 2 utilized a 2 × 2 factorial design to allow investigation into the role of P4 alone, IFNT alone, and P4 and IFNT in combination on endometrial expression of *ALPL* mRNA, TNSALP protein localization, and TNSALP activity. To ascertain the role of P4, the potent anti-progestin RU486 was utilized to prevent transcription of genes regulated by P4-PGR signaling in the ovine uterus [28, 37]. Prior to conceptus attachment, the conceptus is reliant on water, amino acids, hexose sugars, ions, growth factors, hormones, enzymes, cytokines, mitogens, and vitamins (collectively referred to as histotroph) in the uterine lumen that are either secreted by or transported by endometrial epithelia (LE, sGE, and GE) [38, 39]. These components of histotroph are critical in supporting attachment, development, and growth of the conceptus, particularly during the pre- and peri-implantation periods of pregnancy when the ovine conceptus undergoes rapid cell proliferation and migration as it elongates and has high nutritional demands. Alkaline phosphatases are a family of membrane-bound glycoproteins which hydrolyze phosphocompound substrates [including phosphoethanolamine (PEA), inorganic pyrophosphate (PPi), and pyridoxal-5′-phosphate (PLP)] to generate phosphate [3, 4]. The striking apical localization of both TNSALP protein and enzymatic activity, in conjunction with the known secretory functions of the endometrial LE, sGE and GE, suggests that TNSALP could generate phosphate in the epithelial cells to be secreted or transported into the uterine lumen for utilization by the conceptus. In the present study, TNSALP protein appeared to increase in the mid- and deep-endometrial GE in ewes treated with RU486 + P4 + CX compared to the other treatment groups. This finding was accompanied by intense TNSALP activity localized to the apical surface of the deep GE in endometria from ewes treated with RU486 + P4 + CX than that for endometria from ewes in the other treatment groups. In contrast, there was an apparent decrease in TNSALP protein in the deep glands of endometria from ewes treated with RU486 + P4 + IFNT compared to the other treatments investigated. The results of this study suggest that P4 signaling through PGR regulates TNSALP activity in the ovine uterus, independent of IFNT.

Analysis of mRNA expression is an essential component of many utero-placental studies and provides valuable mechanistic insights, although mRNA and protein expression data commonly have poor correlations [40, 41]. The present study and our recent study [6] demonstrated that *ALPL* mRNA, TNSALP protein, and TNSALP enzymatic activity are poorly correlated and highlight that it is essential to not solely rely on mRNA expression data to draw conclusions for this molecule. The TNSALP protein in humans has several post-translational modifications including N-glycosylation five putative sites (Asn140, Asn230, Asn271, Asn303, and Asn430) and an undetermined *O*-glycosylation site [42, 43] which may contribute to differential levels of enzymatic activity. While post-translational modifications of TNSALP have not been reported for sheep, it is reasonable to anticipate that they would be present and may also have an impact on enzymatic activity.

## Conclusions

TNSALP appears to be a regulator of phosphate homeostasis during gestation in ruminants. Utilizing two complementary in vivo approaches, we demonstrated an important role for P4, but not IFNT, in the regulation of TNSALP activity in ovine utero-placental tissues. These data demonstrate a previously unknown regulatory link between P4 and TNSALP that may drive the secretion and/or transport of phosphate into the uterine lumen which is critical for conceptus development.

### Supplementary Information

**Additional file 1: Table S1.** Primer Sequences.

## Data Availability

The datasets used and/or analyzed during the current study are available from the corresponding author on reasonable request.

## Abbreviations

cDNA: Complementary DNA
CO: Corn oil
CX: Control proteins
FGF: Fibroblast growth factor
GCAP: Germ cell alkaline phosphatase
GE: Glandular epithelium
HGF: Hepatocyte growth factor
IAP: Intestinal alkaline phosphatase
IFNT: Interferon tau
LE: Luminal epithelium
P4: Progesterone
PBS: Phosphate buffered saline
PEA: Phosphoethanolamine
PGR: Progesterone receptor
PLAP: Placental alkaline phosphatase
PLP: Pyridoxal-5′-phosphate
PPI: Inorganic pyrophosphate
qPCR: Quantitative polymerase chain reaction
RIgG: Rabbit immunoglobulin
RU486: Mifepristone
sGE: Superficial glandular epithelium
TNSALP: Tissue non-specific alkaline phosphatase
