# Supplementary material for: Progesterone regulates tissue non-specific alkaline phosphatase (TNSALP) expression and activity in ovine utero-placental tissues
Source: J Anim Sci Biotechnol. 2024 Jul 3;15:90. doi: 10.1186/s40104-024-01048-x (PMC11220967; doi:10.1186/s40104-024-01048-x)
Supplement: Supplementary file 1 — Additional file 1: Table S1. Primer Sequences. [file 40104_2024_1048_MOESM1_ESM.docx]

**Table S1** Primer sequences

| **Gene symbol** | **Gene name** | **Accession number** | **Primer sequence (5´→3´)** | | **Tm, ^o^C** | **Amplicon size** |
| --- | --- | --- | --- | --- | --- | --- |
| *ACTB* | Beta-actin | NM_001009784.3 | Fwd | CCACCGCAAATGCTTCTAGG | 60 | 79 |
|  |  |  | Rev | CGTTTTCTGCGCAAGTTAGG |  |  |
| *ALPL* | Tissue non-specific alkaline phosphatase | XM_012152824.3 | Fwd | ACCTTCGCCTACGAACTCTG | 60 | 114 |
|  |  |  | Rev | CCTGCTTTCTCTCTGGAGCC |  |  |
| *B2M* | Beta-2-microglobulin | XM_012180604.2 | Fwd | CATCTTAGCGGTGTGGAGGG | 60 | 72 |
|  |  |  | Rev | TTGTGTGCAAAACACCCTGAC |  |  |
| *GAPDH* | Glyceraldehyde-3-phosphate dehydrogenase | NM_001190390.1 | Fwd | GGGCAGCCCAGAACATCAT | 60 | 112 |
|  |  |  | Rev | CCAGTGAGCTTCCCGTTCAG |  |  |
| *PPIA* | Peptidylprolyl isomerase A | NM_001308578.1 | Fwd | CGCGTCTCTTTTGAGCTGTTT | 60 | 143 |
|  |  |  | Rev | TCACCACCCTGGCACATAAA |  |  |
| *SDHA* | Succinate dehydrogenase complex flavoprotein subunit A | XM_027980212. 1 | Fwd | CATCCACTACATGACGGAGCA | 60 | 90 |
|  |  |  | Rev | ATCTTGCCATCTTCAGTTCTGCTA |  |  |
| *TBP* | TATA-box binding protein | XM_012166509.2 | Fwd | ACCACCGTTTCTTGGTGTCC | 60 | 147 |
|  |  |  | Rev | TGACGTTCACCTTCTTGGCA |  |  |
| *TUB* | Alpha-tubulin | [XM_027967380.2](https://www.ncbi.nlm.nih.gov/entrez/viewer.fcgi?db=nucleotide&id=2062879997) | Fwd | GGTCTTCAAGGCTTCTTGGT | 60 | 94 |
|  |  |  | Rev | CATAATCGACAGAGAGGCGT |  |  |
| *YWHAZ* | Tyrosine 3-monooxygenase/tryptophan 5-monooxygenase activation protein zeta | NM_001267887.1 | Fwd | GACTGGGTCTGGCCCTTAAC | 60 | 72 |
|  |  |  | Rev | GAGAGCAGGCTTTCTCAGGG |  |  |

Experiment 1: *B2M* and *SDHA* were used as reference genes for the endometria, and *PPIA* and *SDHA* used as reference genes for the placentomes. Experiment 2: *TBP* and *YWHAZ* were used as reference genes. Experiment 3: *GAPDH* and *TUB* were used as reference genes for caruncles, and *ACTB* and *SDHA* were used as reference genes for cotyledons
